# Characterization and Cellular Internalization of Spherical Cellulose Nanocrystals (CNC) into Normal and Cancerous Fibroblasts

**DOI:** 10.3390/ma12193251

**Published:** 2019-10-04

**Authors:** Nur Aima Hafiza Shazali, Noorzaileen Eileena Zaidi, Hidayah Ariffin, Luqman Chuah Abdullah, Ferial Ghaemi, Jafri Malin Abdullah, Ichiro Takashima, Nik Mohd Afizan Nik Abd. Rahman

**Affiliations:** 1 Institute of Tropical Forestry and Forest Products (INTROP), Universiti Putra Malaysia, Serdang 43400, Selangor, Malaysia; 2 Department of Cell and Molecular Biology, Faculty of Biotechnology and Biomolecular Sciences, Universiti Putra Malaysia, Serdang 43400, Selangor, Malaysia; 3 Department of Bioprocess, Faculty of Biotechnology and Biomolecular Sciences, Universiti Putra Malaysia, Serdang 43400, Selangor, Malaysia; 4 Department of Chemical and Environmental Engineering, Faculty of Engineering, Universiti Putra Malaysia, Serdang 43400, Selangor, Malaysia; 5 Brain Mapping and Neuroinformatics Unit, Centre for Neuroscience Services and Research (P3Neuro), Health Campus, Universiti Sains Malaysia, Kubang Kerian 16150, Kelantan, Malaysia; 6 Human Informatics Research Institute, National Institute of Advanced Industrial Science and Technology (AIST), Tsukuba, Ibaraki 305-8568, Japan

**Keywords:** fluorescently labelled, cellulose nanocrystals, preparation, cytotoxicity, internalization, shape, endocytosis

## Abstract

The aim was to isolate cellulose nanocrystals (CNC) from commercialized oil palm empty fruit bunch cellulose nanofibre (CNF) through sulphuric acid hydrolysis and explore its safeness as a potential nanocarrier. Successful extraction of CNC was confirmed through a field emission scanning electron microscope (FESEM) and attenuated total reflection Fourier transmission infrared (ATR-FTIR) spectrometry analysis. For subsequent cellular uptake study, the spherical CNC was covalently tagged with fluorescein isothiocyanate (FITC), resulting in negative charged FITC-CNC nanospheres with a dispersity (Ð) of 0.371. MTT assay revealed low degree cytotoxicity for both CNC and FITC-CNC against C6 rat glioma and NIH3T3 normal fibroblasts up to 50 µg/mL. FITC conjugation had no contribution to the particle’s toxicity. Through confocal laser scanning microscope (CLSM), synthesized FITC-CNC manifested negligible cellular accumulation, indicating a poor non-selective adsorptive endocytosis into studied cells. Overall, an untargeted CNC-based nanosphere with less cytotoxicity that posed poor selectivity against normal and cancerous cells was successfully synthesized. It can be considered safe and suitable to be developed into targeted nanocarrier.

## 1. Introduction

Cancer is a group of diseases characterized by the unstoppable proliferation of abnormal cells. Until today, the cure for cancer has yet to be found. However, several lines of treatment regimens have been established to eradicate and slow down the cancer progression. There are three classes of conventional cancer therapy, which are surgery, chemotherapy, and radiotherapy [1]. For cases involving malignancies that can be easily distinguished from the healthy normal cells, surgery is the best option. However, for cancers that are not suitable for the surgical procedure, chemotherapy and radiotherapy are the possible alternatives. Nevertheless, not all anti-cancer drugs and radiation therapy work effectively in individuals. Resistance towards conventional therapies is often reported besides the emergence of secondary tumours. One way to explain these occurrences is that cancer cells are genetically heterogeneous and capable of synthesizing a drug efflux pump [2]. Hence, the same chemotherapy drug may not be as effective as it is in some individuals. Besides, due to poor selectivity of treatment towards cancerous cells, a high dosage of chemotherapy is often administered, leading to cytotoxic effect to the surrounding healthy cells [3]. This, in turn, would give rise to side effects, such as nausea, vomiting, and hair loss. Today, the idea of personalized anti-cancer drugs has captured many researchers’ attention. In reality, this may take a very long time to be developed and is cost-consuming. 

While the discovery of the new anti-cancer drugs is still on-going, it was believed that the efficacy of chemotherapy may be improved through the active delivery approach. As mentioned, cancer patients may respond to the established chemotherapy at different extent. For those who showed little effect may need higher regime dose to amplify the drug effectiveness [3]. However, high dose treatment may cause a cytotoxic effect on healthy cells [4]. Therefore, by applying active or targeted drug delivery, it was proposed that the regime dose can be lowered as selective uptake by cancer cells is expected. Moreover, if this approach is a success, patient compliance could be improved since lesser side effects are projected by the healthy cells [5]. 

In tailoring a good drug carrier, natural polymeric nanoparticles are more preferred due to its extremely small size, high drug loading capacity [6], longer bioavailability [5], less to non-toxic to cells, biocompatibility, and availability for versatile surface functionalization and modifications [7]. For these reasons, nanocellulose has received enormous interest for its potential application as drug nanocarrier. Cellulose is the most abundant natural polymer on Earth since it is the primary constituent of the plant cell wall. For the past decades, with the advancement of nanobiotechnology, cellulose nanocrystals (CNC) have been extensively prepared and studied for its potential applications in various fields including biomedical engineering [8,9,10]. One of the most explored fields of study is the utilization of CNC as nanocarrier. Hieratically, cellulose is made up of β-glucose monomers that are linked together at the first and the fourth carbon atoms by a b-glycosidic bond. Approximately 36 individual polymer chains interact with each other through inter- and intra-molecular hydrogen bonding to form the elementary CNC. These nanocrystals are then packed together and undergo spinning at the site of synthesis, into larger and longer cellulose nanofibres (CNF) which consist of the highly ordered crystalline and the disoriented amorphous regions [11]. Upon removal of amorphous cellulose regions in CNF through acid hydrolysis, individual nanocrystals will be released. 

It was also fundamental to understand the interrelationship between the nanocarrier’s physicochemical properties and its biological behavior towards cells. In general, it was widely known that factors such as the shape, size and surface properties of nanoparticles will determine its cellular internalization. In term of shape, spherical nanoparticles were found to show enhanced cellular uptake compared to nanorod, nanocube, and nanodisk particles [12]. This is because, compared to other non-spherical nanoparticles, the geometry of the nanosphere makes it more readily prone to have a higher length normalized curvature. In order to achieve similar length normalized curvature, the non-spherical nanoparticles need to interact with the cell membrane at specific contact orientation [13]. Since this occurrence happens at random, thus non-spherical nanoparticle exhibit cellular internalization at a lesser extent compared to the nanosphere. It was also suggested that nanoparticles with the size of ≤ 100 nm would escape opsonization by immune cells, limiting immediate clearance by the lymphatic system [7]. This thus leads to longer bioavailabity. In addition, for targeted delivery purpose, the untargeted nanocarrier should exhibit poor cellular internalization since the targeted property should only be acquired upon surface functionalization with selective ligands [14].

Although numerous studies have evaluated the cytotoxicity and cellular uptake of acid hydrolysed-CNC, however, most of them only focused on the rod or needle-like shaped CNC [14,15,16]. Only one study has successfully synthesized spherical CNC (directly from commercialized trimethylsilylcellulose instead of acid-hydrolysed-CNC) and investigated its cytotoxicity and cellular accumulation [17]. In the same study, authors reported that the untargeted spherical CNC, conjugated with fluorescein isothiocyanate (FITC), manifested a rapid cellular uptake via endocytosis. For this reason, it was believed that the untargeted spherical FITC-CNC produced by the team may not be suitable to be developed into a safe targeted nanocarrier. Therefore, present study attempted to improvise the synthesis method of spherical FITC-CNC using acid-hydrolyzed-CNC and study its cytotoxicity and cellular internalization into C6 and NIH3T3 fibroblast cells for potential anti-cancer drug nanocarrier application.

## 2. Materials and Methods 

### 2.1. Materials

CNF derived from oil palm empty fruit bunch (OPEFB) was purchased from a commercial source (ZOEPNANO Sdn. Bhd., Selangor, Malaysia). Sulphuric acid (99%, Classic Chemicals, Selangor, Malaysia), sodium hydroxide (Ajax Finechem Pty Ltd., Taren, Australia), sodium borate decahydrate (Bio Basic, Ontario, Canada). Ammonuim hydroxide, epichlorohydrin, fluorescein isothiocyante (FITC) powder, ethylene glycol tetraacetic acid, SnakeSkinTM dialysis tubing with molecular weight cut off (MWCO) 10kDa, 3-(4,5-dimethyl-thiazol-2-yl)-2,5-diphenyl-tetrazoliumbromide (MTT), 4′,6-diamino-2-phenylindole (DAPI), paraformaldehyde powder, Dulbecco’s Modified Eagle’s Medium (DMEM), Roswell Park Memorial Institute (RPMI) and Accutase^®^ solution were purchased from Sigma-Aldrich, St. Louis, MO, USA, while dimethyl sulfoxide (DMSO), Triton X-100, Fetal Bovine Serum (FBS) and Penicillin-Streptomycin solution were from Thermo Fisher Scientific, Pittsburgh, PA, USA. CYTO-ID^®^ Red long-term cell tracer kit was from Enzolife, PA, USA and Phosphate Buffer Saline (PBS) was from TBUSA, Mountain View, CA, USA. 

### 2.2. CNC and FITC-CNC Synthesis

CNC was isolated via sulphuric acid hydrolysis treatment of CNF, leading to a negatively charged CNC, as described in a previous protocol with slight modifications [18]. Briefly, 1 g CNF was dispersed in 20 mL 65% (w/w) sulphuric acid solution and heated at 45 °C for 2 h. The reaction was quenched by diluting the suspension 5 times with chilled distilled water. The resulting CNC was rinsed with 0.1 M sodium hydroxide solution to facilitate the removal of acid. Then, CNC was thoroughly washed through repeated centrifugation and redispersion in deionized water before being dialyzed until constant pH was achieved. Subsequent ultrasonication procedure was performed for 10 min at 40% output in an ice bath to ensure dispersion prior to freeze-drying for subsequent characterization purpose. 

For the downstream cellular uptake study, FITC was conjugated onto the CNC following literature procedure [19]. One g of CNC was mixed with 130 mL of 1 M sodium hydroxide solution and epichlorohydrin (6 mmol/g CNC). The mixture was heated at 60 °C for 2 h and dialyzed until the pH was below 12. After adjusting the pH to 12, 5 mL of 30% (*v*/*v*) ammonium hydroxide solution was added, and the mixture was heated as previous. The suspension was dialyzed until neutral. Next, 240 mL of 50 mM sodium borate buffer and 2 mg of FITC were added and allowed to react under constant stirring overnight in the dark at room temperature. Ultrasonication was performed as previously described before the suspension was dialyzed until no trace of FITC was detected in the dialysis effluent. The amount of FITC moieties mounted per g CNC was deduced using UV-Vis spectrometry at 492 nm. Note that all dialysis steps throughout the experiment were performed using regenerated cellulose tubular membrane (MWCO 10kDa).

### 2.3. Characterization of CNF, CNC, and FITC-CNC

#### 2.3.1. Field Emission Scanning Electron Microscopy (FESEM)

FESEM was used to study the surface morphology of CNF and CNC to confirm the successful isolation of CNC. A thin layer of the sample was mounted separately onto a carbon-coated metal holder and treated with gold to ensure good conductivity during imaging. FESEM micrographs were taken at a few magnifications with an accelerating voltage of 5 kV using JEOL (Tokyo, Japan) JSM7600F Field Emission SEM.

#### 2.3.2. Attenuated Total Reflection Fourier Transmission Infrared (ATR-FTIR)

ATR-FTIR analysis was carried out to detect the changes in chemical composition between CNF, CNF, and FITC-CNC to further confirm CNC isolation and FITC conjugation. FTIR spectra of the freeze-dried samples were recorded within the range of 400–4000 cm^−1^ using the Thermo Fisher Scientific (Pittsburgh, PA, USA) Nicolet iS10FT-IR Spectrometer.

#### 2.3.3. Zeta Potential and Polydispersity Index 

The zeta potential of CNC and FITC-CNC in aqueous medium of pH 6.5 at 25 °C was determined using Malvern 3600 Zetasizer NanoS (Malvern Instrument, Malvern, UK) in combination with Dispersion Technology Software (DTS, Malvern, UK). The same instrument was used to measure the dispersity (Ð) of both samples in the same condition. 

#### 2.3.4. Atomic Force Microscopy (AFM) 

AFM was conducted to study the morphology and dimension (length and diameter) of CNC and FITC-CNC. Diluted never-dried CNC and FITC-CNC were dropped onto a glass slide and allowed to air dry. Samples were scanned at room temperature, in tapping mode with OMCL-AC160TS standard Si probes (tip radius < 10 nm, spring constant = 2.98 N/m, resonant frequency = ~310kHz) using the Dimension Edge with High-Performance AFM (Bruker, Sant Barbara, CA, USA) equipment. AFM micrographs were analyzed using Bruker Nanoscope analysis software (Version 1.7) operated using Peak/Force tapping mode with one controller (Nanoscope V from Bruker) for evaluating the length and width of the samples.

### 2.4. Cytotoxicity and Cell Viability Assay

#### 2.4.1. Cell Culture

Two types of mammalian cell lines, C6 (rat glioma) and NIH3T3 (normal murine fibroblast), were used for this study. Both cells were obtained from American Type Culture Collection (ATCC, Manassas, VA, USA). Cells were cultured in DMEM and RPMI, respectively, supplemented with 10% FBS, 100 U/mL Penicillin and 100 µg/mL Streptomycin and were incubated in a 5% CO_2_ incubator at 37 °C and were passaged using Trypsin-EDTA every 3–4 days.

#### 2.4.2. MTT Assay

The MTT assay was performed as previously described [20,21]. In brief, cells were seeded at 1.5 × 10^4^ cells per well in a 96-well plate and were incubated overnight to allow cell attachment. A stock suspension of 100 µg/mL of CNC and FITC-CNC were prepared, sonicated for 10 min, and filtered through 0.45 µm filter for sterilization. After replacing the medium with a fresh one, cells were treated with CNC and FITC-CNC at 50 µg/mL, 25 µg/mL, 12.5 µg/mL, 6.25 µg/mL, 3.13 µg/mL, and 1.56 µg/mL. Untreated cells served as control. After 24 h incubation, MTT reagent (0.5 mg/mL) was added into each well, and cells were let to further incubated for 4 h to allow formazan crystals formation. Next, 170 µL of the medium from each well was discarded, and 100 µL DMSO was added to solubilize the crystals. Cell viability was deduced from the absorbance reading at 570 nm using an ELISA plate reader, following the formula:

Cell viability (%) = (A(x)/A(o)) × 100%,(1)

where A(x) is the absorbance reading at certain concentration of treatment, while A(o) is the absorbance reading of untreated culture [22]. The assay was performed in triplicate for each cell line. 

### 2.5. Cellular Internalization Study

Following the manufacturer’s protocol, C6 cells and NIH3T3 cells were stained for their cytoplasm using the Cyto-ID Red long-term cell tracer kit (Enzolife) before 1 × 10^5^ cells were seeded into each confocal dish (20 mm diameter, glass-bottom). Cells were incubated overnight for cell attachment. Next, cells were treated with 50 µg/mL FITC-CNC for 4 h before being fixed and permeabilized using 3.8% paraformaldehyde and 0.1% Triton X-100, respectively. The cell nucleus was stained with DAPI. After washing with PBS, cells were viewed under the Nikon A1+ Confocal Laser Scanning Microscope (Nikon Instrument Inc., Melville, NY, USA) using DAPI, tetramethylrhodamine isothiocyanate (TRITC), and FITC filters. Cells treated with equivalent FITC served as control.

### 2.6. Statistical Analysis

Statistical analysis and graphs were performed and generated using KaleidaGraph Synergy Software (Version 4.5.2).

## 3. Results and Discussion

**Figure 1:**
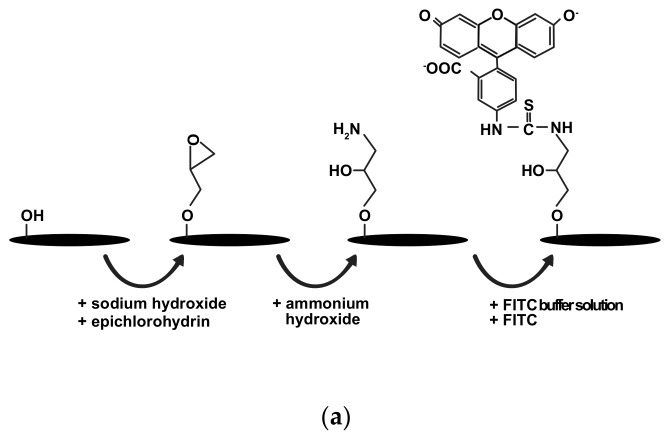

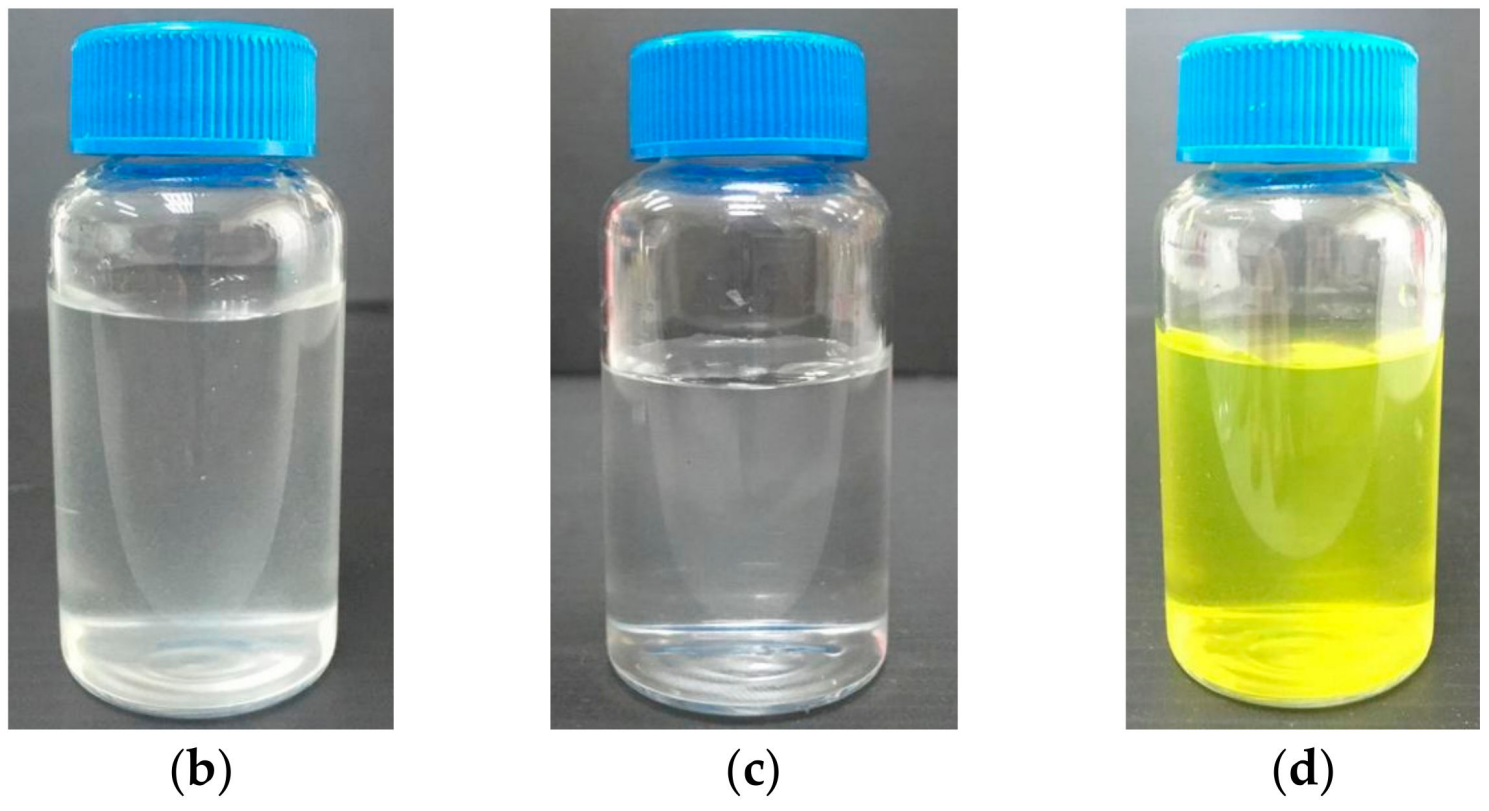
Photographs of aqueous suspension of (**a**) schematic diagram illustrating the reaction of conjugating fluorescein isothiocyanate (FITC) onto cellulose nanocrystals (CNC). Photographs of aqueous suspension of (**b**) 500 μg/mL cellulose nanofibre (CNF), (**c**) 100 μg/mL CNC, and (**d**) 100 μg/mL FITC-CNC.

Sulphuric acid hydrolysis was the most common protocol used to extract CNC from CNF. In this process, β-1,4-glycosidic bonds and intermolecular hydrogen bonds between polymer chains were broken to release the individual crystals [23]. The subsequent FITC conjugation onto the crystals was done through the one-pot procedure to mount fluorescent moieties for internalization study. The conjugation reaction can be summaries as illustrated in Figure 1a. Briefly, epichlorohydrin was grafted onto CNC in sodium hydroxide solution, forming epoxy-activated CNC. This process was followed by epoxy ring-opening with aqueous ammonium hydroxide to create primary amine groups, to which FITC could be covalently attached [24]. Finally, FITC was let to react with the primary amine groups in FITC buffer solution to form FITC-CNC. Figure 1b–d showes the physical appearance of aqueous CNF, CNC, and FITC-CNC suspensions, respectively. CNF was colourless but slightly turbid, and CNC appeared colourless and slightly opaque, while FITC-CNC was yellow and slightly opaque, similar to as reported in the literature [25]. 

### 3.1. Nanocellulose Synthesis and Characterization

#### 3.1.1. FESEM

**Figure 2:**
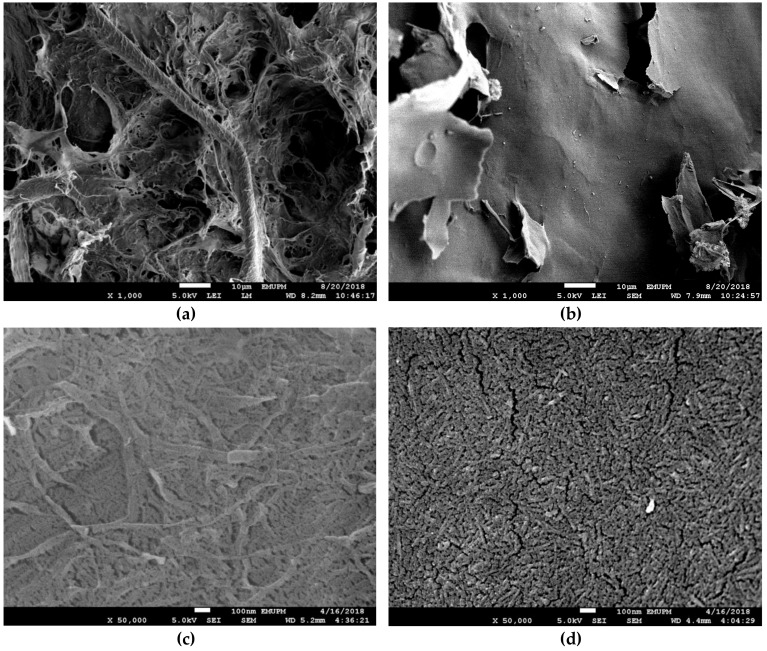
Field emission scanning electron microscope (FESEM) micrograph of 2 wt% freeze dried (**a**) CNF at 5000 magnification, (**b**) CNC at 5000 magnification, (**c**) CNF at 50,000 magnification, and (**d**) CNC at 50,000 magnification.

The surface morphology of the freeze-dried CNF and CNC was compared through FESEM imaging to confirm the successful extraction of CNC. As presented in Figure 2a, it was observed that CNF twined together without any uniformity, forming a rough surface with tortuous appearance. Meanwhile, CNC in Figure 2b was dried homogeneously into smooth thin lamellae. At higher magnification, it revealed that individual CNF in Figure 1c was filamentous and branchy. However, individual CNC in Figure 1d was relatively short and similar in size.

Freeze drying is a three-step process that eliminates water molecules from a sample suspension. The first step was to freeze the samples at −80 °C, where water molecules were separated from the nanocellulose particles by forming ice crystals. These ice crystals were sublimated during the primary drying phase while the remaining nonfreezing bound water was removed in the secondary drying phase. These events caused an increase in the local nanocellulose concentration, making the individual fibre/crystal to have physical contact with one another [26].

The appearance of freeze-dried foam was known to be influenced by the particle size and suspension concentration [27]. While the later variable was kept constant at 2 wt%, the difference in surface morphology or CNF and CNC can be inferred to the difference in the particle size. The individual fibre in CNF was long and filamentous. When water is removed, they agglomerated randomly and laterally through diffuse forces or hydrogen bonding, or both [26]. After elimination of the amorphous region in CNF via acid hydrolysis, the resulting crystals were shortened to nanosized length. Upon dispersion in water, CNC self-assembled into chiral nematic or cholesteric structure over time [28]. This uniform arrangement is characterized by stacked planes of CNC that aligned along with a director, which rotated about a perpendicular axis from one plane to another [29]. Since each plane is parallel to one another, these stacks of CNC can be compressed uniformly into a thin smooth-surfaced layer when water is removed [27]. Although it was nearly impossible for CNF to achieve similar morphology due to its high dispersion component of surface energy [30], however, previous study has shown that an evener surface morphology can be obtained by dramatically lower the CNF concentration subjected to lyophilization [27]. This action will reduce the CNF dispersion component of surface energy, making the individual fibre in the suspension to be distributed singly more readily [31]. 

#### 3.1.2. ATR-FTIR

**Figure 3:**
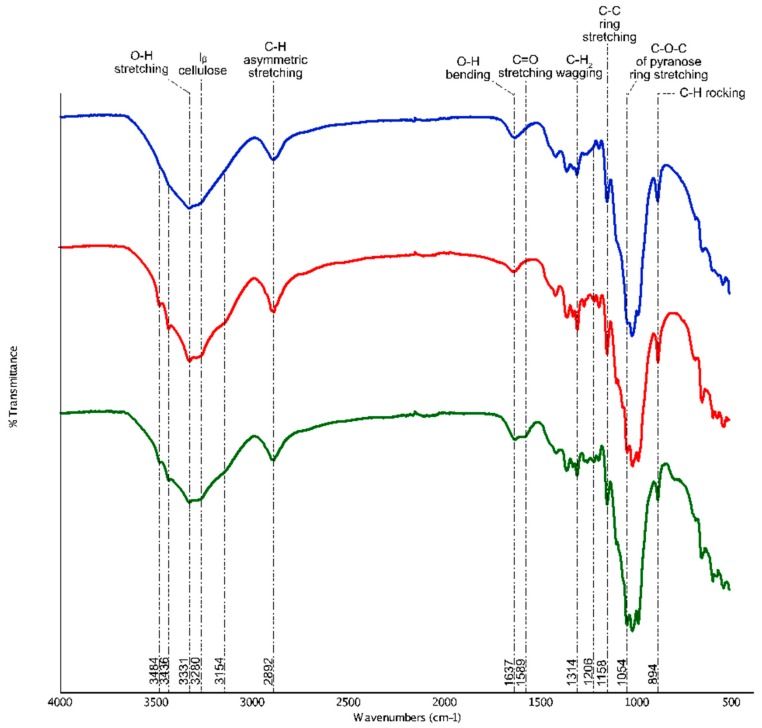
Attenuated total reflection Fourier transmission infrared (ATR-FTIR) spectra of CNF (blue), CNC (red), and FITC-CNC (green).

The fingerprints of the functional groups contained in CNF and CNC were detected through FTIR spectroscopy, as shown in Figure 3. In general, CNF and CNC shared most intensity peaks related to cellulose. These peaks are around 3331 cm^−1^, 2892 cm^−1^, 1637 cm^−1^, 1314 cm^−1^, 1158 cm^−1^, 1054 cm^−1^, and 894 cm^−1^, which are responsible for the stretching vibration of O–H, asymmetric stretching of C–H, O–H bending in water, C–H_2_ wagging vibration, C–C ring stretching, the stretching vibration of C–O–C in pyranose ring, and C–H rocking, respectively [32,33,34]. Eight hundred and ninety-four cm^−1^ was also described to represent the glycosidic deformation, which was the characteristic of β-glycosidic linkage between glucose monomers in cellulose [35,36].

The first dissimilarity between CNF and CNC that can be observed from the spectra was the emergence of four distinct peaks around 3484 cm^−1^, 3436 cm^−1^, 3280 cm^−1^, and 3154 cm^−1^ in CNC, which were absent or marked very faintly in CNF. These pikes were associated with cellulose type II structure [37,38]. It was widely known that cellulose type I is the native structure of cellulose in nature. In this confirmation, glucose ring of the adjacent β-1,4-glucan chains of cellulose crystals are arranged in a parallel manner where they are held together by intermolecular hydrogen bonds between C3OH–OC6 (hydroxyl group on the third carbon chain to the oxygen atom on carbon sixth). In the present experiment, to facilitate the removal of residual sulphuric acid traces, CNC obtained post-hydrolysis was rinsed with 0.1 M sodium hydroxide solution. During this process, apart from displacing the sulfate groups from cellulose backbone, sodium ions also interacted with C6O to form Na-cellulose I intermediates, similar to the mercerization process. This swelling of native cellulose resulted in the breakage of intermolecular hydrogen bonds that separate the adjacent chains apart. Recovering of this breakage through successive washing and dialysis against distilled water, however, has irreversibly converted cellulose I to cellulose II arrangement [29,39].

In regenerated cellulose II structure, as represented by the FTIR spectra of CNC, the chemical alteration only involved the free hydroxyl groups that ranged between 3100–3700 cm^−1^. According to the literature, peaks at 3484 cm^−1^ and 3436 cm^−1^ represented the intramolecular hydrogen bonding between C3OH–OC6 (minor component) and C3OH–OC5 (major component), respectively [27,37,39,40]. Peaks at 3280 cm^−1^ and 3154 cm^−1^, on the other hand, signified the new intermolecular bonding pattern between C2OH–OC6 and C6OH–OC2, correspondingly. Although CNF and CNC shared similar peak around 3331 cm^−1^, however, this wavenumber was designated for different fingerprints. For cellulose I in CNF, peak 3331 cm^−1^ signified the intramolecular hydrogen bonding between C6OH–OC2 or C2O–OC6, or both. While in cellulose II CNC, peak at 3331 cm^−1^ indicated the intermolecular hydrogen bond between C2OH–OC2 or C6OH–OC6, or both [37]. In addition, the minute transmittance observed at 3280 cm^−1^ in cellulose I CNF was proposed to be detected as the monoclinic cellulose Iβ subtype identification [41]. However, a similar peak was recognized as the intermolecular bonding between C2OH–OC6 in cellulose II CNC, as mentioned earlier [37,40]. Briefly, the introduction of sodium ions had caused the CNC to be arranged in cellulose II manner. Besides, the free hydroxyl groups present in the sample has been intensified (FTIR cumulative peak intensity: CNF = 74.7, CNC = 163.56) through transient swelling of CNC, making the synthesized CNC more reactive for any downstream chemical modifications [11]. 

Another worth mentioning difference between CNF and CNC that can be observed through FTIR spectra was the peak intensity at 1637 cm^−1^, which represented the O–H bending of absorbed moisture in the sample [42]. In CNF, this peak’s intensity was relatively higher than CNF compared to CNC (FTIR peak intensity: CNF = 91.25, CNC = 89.11). Notably, the presence of abundant amorphous region CNF which was proven to contribute to the wettability and swelling of CNF upon interacting with water molecules in a computational modelling study [43]. Since CNC have a greatly reduced amorphous cellulose, therefore, it was less susceptible to form hydrogen bonding with water. Next, the presence of a peak at 1206 cm^−1^ in CNC can be associated with the residual sulfate groups from sulphuric acid hydrolysis. Overall, it can be concluded that CNC has been successfully extracted from CNF via acid hydrolysis along with the sharpening of peaks from 3100–3700 cm^−1^ in CNC [44]. 

The positive conjugation of FITC moieties onto CNC was also confirmed through FTIR spectroscopy. As shown in Figure 2, a peak at 1589 cm^−1^ was marked in FITC-CNC which represented C=O stretching vibration of protonated carboxyl groups in FITC [45]. Besides, a remarkable reduction of the free hydroxyl groups in FITC-CNC was also observed, (FTIR cumulative peaks intensity: CNC = 163.56, FITC-CNC = 84.97) since FITC conjugation extended from the hydroxyl groups of CNC. Also, based on the UV-Vis spectrometry analysis (not shown), it was deduced that 0.073 mmol of FITC moieties were covalently mounted per gram CNC which was more than the amount reported in previous literature [24,45]. In terms of surface charge, as presented in Table 1, it was found that conjugation of FITC had altered the zeta potential of CNC from −38.6 mV to −17.7 mV while the Ð improved from 0.703 to 0.371, which was considered acceptable for the delivery application [46].

**Figure 4:**
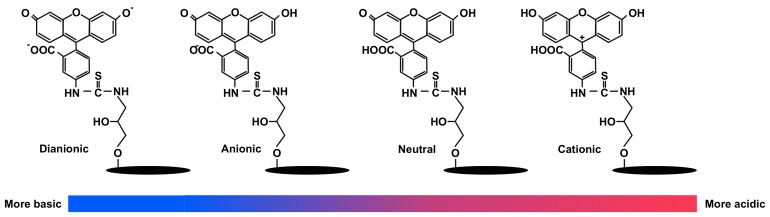
Different isoforms of FITC-CNC that present predominantly at different pH condition.

Zeta potential is a parameter used to study the net surface charge of particular nanoparticles in a medium. The measurement was recorded at pH 6.5 to mimic the physiological pH condition of the studied cell culture [15]. Based on the figure, it can be said that conjugation of FITC onto CNC had increased its zeta potential to be less negative. This occurrence can be justified by the protonation of FITC isomers at slightly acidic condition [47]. As illustrated in Figure 4, depending on pH, FITC exists in four different protonic forms, which are dianionic, anionic, neutral, and cationic. While cationic and neutral isoform concentration is negligible at pH > 4 [48], hence, it can be ruled out that the increase in zeta potential was contributed by the formation of more anionic FITC through dianionic isoform protonation. As the pH becomes more acidic, the zeta potential of FITC-CNC become more positive as more dianionic FITC-CNC become protonated. In fact, the shift in zeta potential due to FITC protonation has been evident to overcome the electrostatic repulsive force between particle surface and the cell membrane, thus promoting the rod-shaped FITC-CNC internalization into HEK 293 and Sf9 at pH 5 [15].

#### 3.1.3. AFM

**Figure 5:**
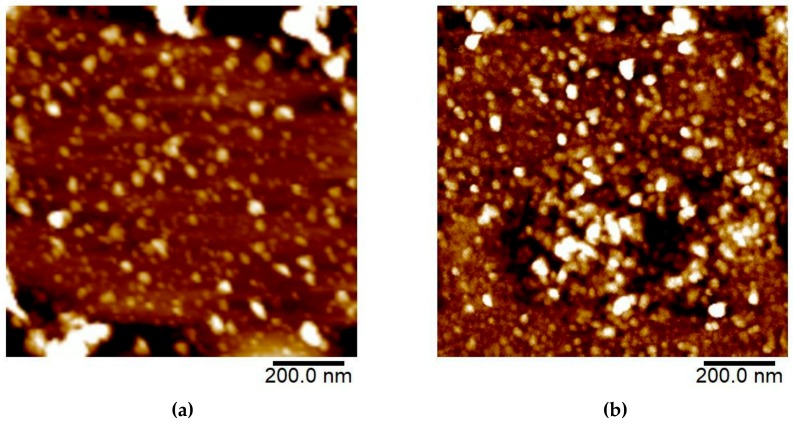

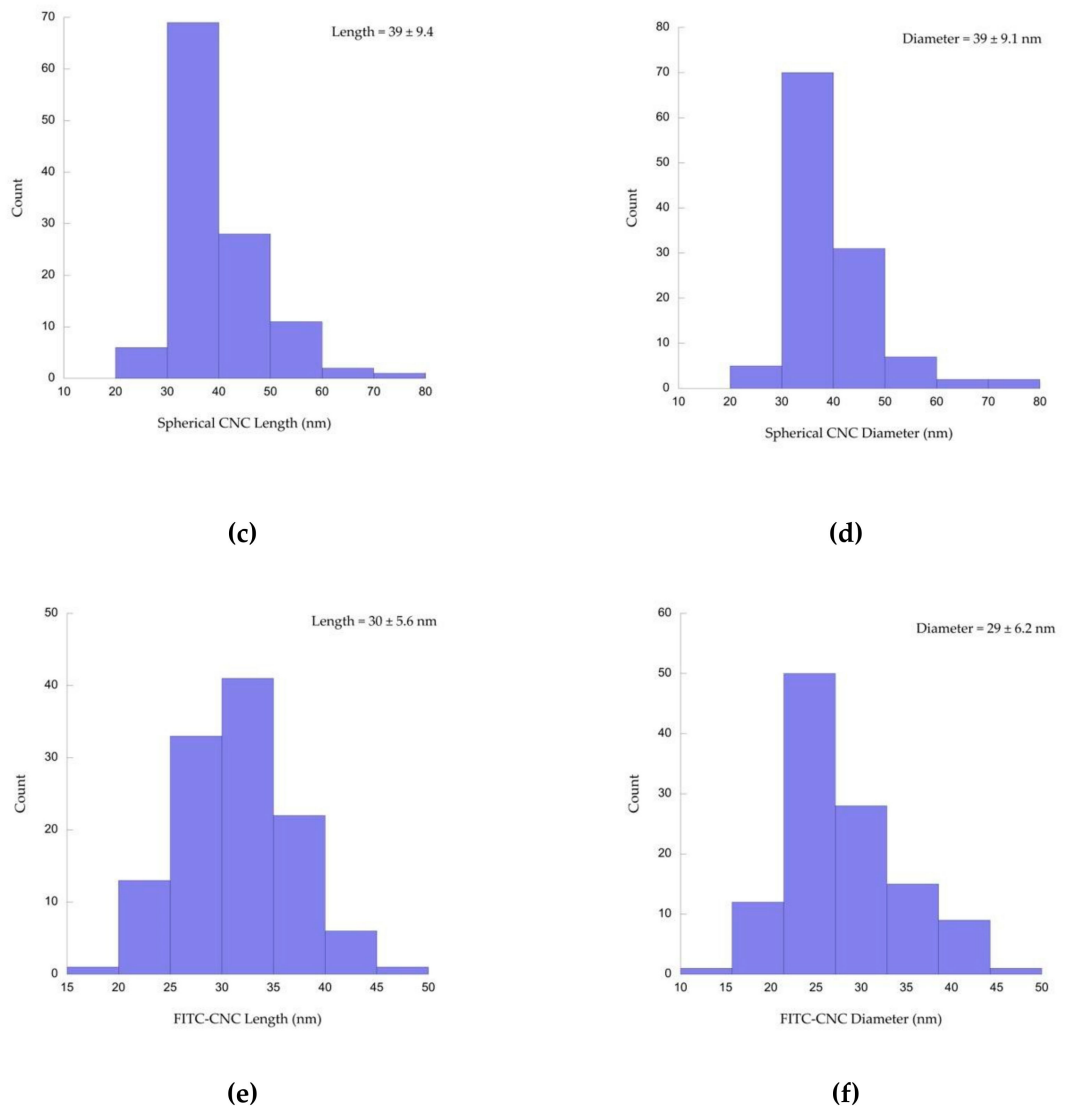
Atomic Force Microscopy (AFM) micrograph of (**a**) CNC and (**b**) FITC-CNC. Histogram of (**c**) spherical CNC length, (**d**) spherical CNC diameter, (**e**) spherical FITC-CNC length, and (**f**) spherical FITC-CNC diameter with their respective averages.

Diluted CNC and FITC-CNC were characterized for their morphology, length, and diameter through AFM. From the AFM micrographs in Figure 5a,b, it was observed that both samples formed nanospheres. Using the specified software, it was deduced that the mean length and diameter of the spherical CNC was 39 ± 9.4 nm and 39 ± 9.1 nm, correspondingly (Figure 5c,d). Meanwhile, the mean length and diameter for the spherical FITC-CNC was 30 ± 5.6 nm and 29 ± 6.2 nm, respectively (Figure 5e,f). The aspect ratio (length/diameter) for both spherical CNC and FITC-CNC was 1.0 ± 0.14 individually. Although the aspect ratio of spherical CNC and FITC-CNC remained unchanged, however, CNC was relatively larger than FITC-CNC. It was also worth mentioning that a small portion of ribbon-shaped CNC with an average length, diameter, and aspect ratio of 153 ± 66 nm, 4.2 ± 1.5 nm, and 4.9 ± 2.8 was observed in the CNC sample (Supplementary Figure S1). 

CNC with cellulose II arrangement has been previously reported to adapt a few morphologies, depending on its preparation conditions (alkaline solution concentration, ultrasonication, sulphuric acid concentration, hydrolysis duration, etc.). For instance, a ribbon-shaped cellulose II with the length and diameter of 153 ± 66 and 4.2 ± 1.5 nm has been isolated from cotton cellulose through sulphuric acid hydrolysis at 66 wt% final acid concentration with reaction duration longer than 60 min [49]. Meanwhile, cellulose II granules with the size of 150–200 nm were observed after treating cellulose I with sodium hydroxide solution at concentrations higher than 8 wt% [18]. In the same study, the authors also highlighted the coexistence of the needle-like cellulose I and cellulose II nanosphere at transition concentrations of sodium hydroxide solution. Based on the particles’ shape observed, it can be assumed that the ribbon-shaped CNC may rise from the harsh hydrolysis condition (65% sulphuric acid, 2 h) while the spherical CNC formed upon rinsing with sodium hydroxide solution. The 0.1 M sodium hydroxide used, however, may not be sufficient to convert the ribbon-shaped CNC to nanospheres. 

Nevertheless, after FITC conjugation, all particles were witnessed to be fully transformed into spherical cellulose II due to excess sodium hydroxide solution that has been introduced to swell the particles. With the aid of the mechanical shear from ultrasonication, intermolecular and intramolecular hydrogen bonds were broken down by cavitation, releasing the shortened and spherical FITC-CNC with the average size of 30 nm [50]. This was significantly smaller than those produced from the trimethylsilylcellulose [17].

### 3.2. Nanocellulose Cytotoxicity

**Figure 6:**
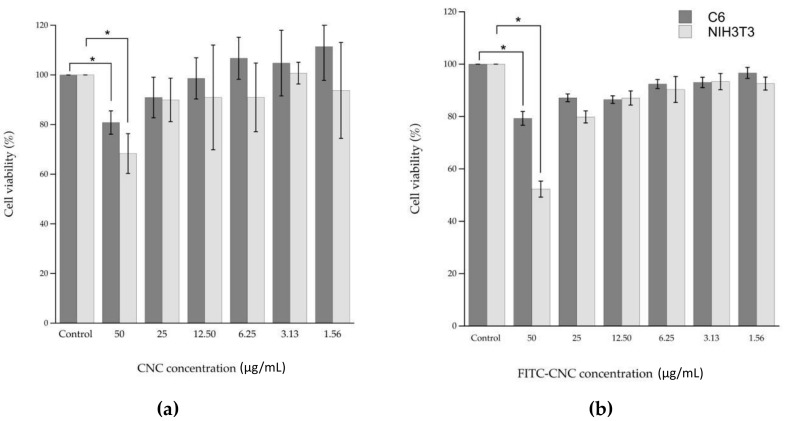
Cell viability (%) of C6 and NIH3T3 after being treated with (**a**) CNC and (**b**) FITC-CNC at 50 μg/mL, 25 μg/mL, 12.5 μg/mL, 6.25 μg/mL, 3.13 μg/mL, and 1.56 μg/mL for 24 h. * indicates that the *p*-value < 0.05.

The cytotoxicity of CNC and FITC-CNC was measured using the MTT assay. In this analysis, MTT salt served as a substrate for intracellular dehydrogenases to form formazan crystals. In theory, the higher the amount of formazan formed, the higher the number of viable cells. Thus, the lower the cytotoxic effect of the sample [22]. As presented in Figure 6a,b, the untagged CNC and FITC-CNC exhibited a dose-dependent reduction in cell viability. It was also found that only at 50 μg/mL the cell viability decreased significantly for both samples in both cell lines. At the remaining concentrations, treatments were less to non-toxic. Based on the results, it can be illustrated that there was no substantial difference in the cell viability reduction induced by both samples at the same concentrations. Hence, FITC-conjugation did not contribute to the particle’s cytotoxicity. In addition, the detrimental effect of the negatively charged nanocelluloses on cell viability at 50 μg/mL can be attributed to its tendency to interfere with the cell spreading [15]. 

### 3.3. FITC-CNC Cellular Internalization

**Figure 7:**
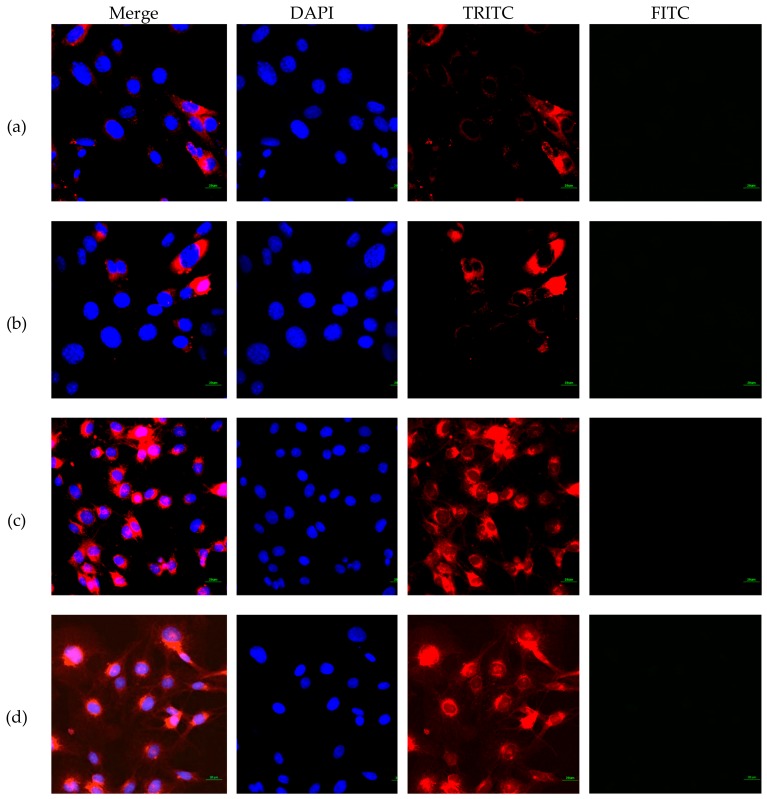
Confocal images of (**a**) FITC-treated NIH3T3, (**b**) FITC-CNC-treated NIH3T3, (**c**) FITC-treated C6, and (**d**) FITC-CNC-treated C6 cells viewed under 4′,6-diamino-2-phenylindole (DAPI), tetramethylrhodamine isothiocyanate (TRITC), and FITC filters. Scale bar = 20 μm.

The internalization of FITC-CNC into C6 and NIH3T3 cells after 4 h incubation was visualized under the confocal laser scanning microscope. Since the cell viability reduction was less than 50%, hence the maximum concentration of 50 μg/mL was used in the internalization study. As presented in Figure 7, a negligible detection of the green fluorescence was observed under FITC filter, suggesting that FITC-CNC exhibited poor cellular uptake into both normal and cancerous cell lines.

Nonphagocytic cells such as C6 and NIH3T3 take up nanoparticles mainly through receptor-mediated or non-specific adsorptive endocytosis [19]. While the former was not applicable for the present study, the non-specific endocytosis of CNC was advocated to be governed by several factors, including particles’ surface charge [15], shape [17], and hydrophobicity [51,52]. Similar to other nanomaterials, the influence of surface charge affects particle adhesion onto the cell surface. For the negatively charged CNC, cellular accumulation was inhibited due to electrostatic repulsive force between the CNC surface and the negative charge cell membrane. As a result, CNC failed to adhere to the cell surface to initiate membrane wrapping. On the contrary, positively charged CNC can be adsorbed onto the cell surface and be taken up by cells readily [15]. 

Previously, the geometry of FITC-CNC was advocated to govern its internalization into human foreskin fibroblasts through endocytosis. While most study involving FITC-CNC produced rod-like shape particles [14,15,16,19], Liebert and colleagues successfully synthesized spherical FITC-CNC directly from commercialized trimethylsilylcellulose [17]. After 2 h incubation, the human foreskin fibroblast used in the study was found to be highly loaded with FITC-CNC nanospheres, suggesting a rapid internalization through endocytosis. However, contradicting observation was obtained in the present study where negatively charged spherical FITC-CNC did not show any noticeable uptake into C6 and NIH3T3 under confocal imaging. 

**Figure 8:**
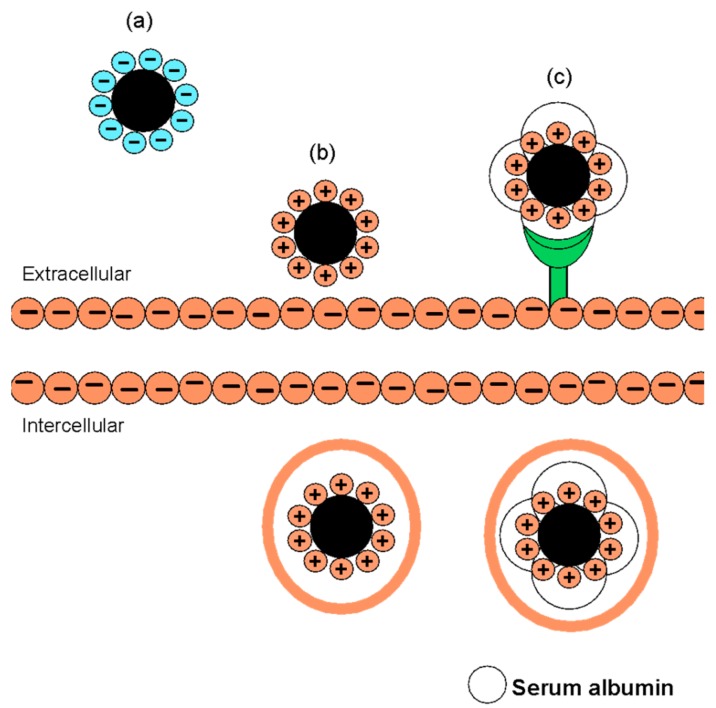
Illustration of the possible uptake mechanisms of FITC-CNC nanospheres with different surface charges. (**a**) Negatively charged FITC-CNC, which fails to overcome the electrostatic repulsive force against the cell membrane, could not adhere onto the cell surface to initiate membrane wrapping. (**b**) Positively charged or reasonably less negative FITC-CNC could adhere onto the cell surface and is taken up through non-specific adsorptive endocytosis. (**c**) Positively charged or reasonably less negative FITC-CNC could also possibly enter intracellular matrix through receptor-mediated endocytosis by forming bio-corona.

Since no data on zeta potential was provided and no acid hydrolysis reaction was done, it was sensible to speculate that FITC-CNC nanospheres prepared by the team may pose a reasonably less negative surface charge, most likely between −5 mV to −15 mV [53], that allows particle adhesion onto the cell surface. Hence, the particle can be taken up through the non-specific adsorptive endocytosis, as illustrated in Figure 8. Another possible route of internalization for the spherical FITC-CNC was through receptor-mediated endocytosis. In in vitro study, the unintended formation of bio-corona could occur, depending on the particle surface charge [54]. Recently, a significant interaction between positive charge CNC with bovine serum albumin based on charge neutralization has been reported [55]. Formation of protein corona around negatively charged nanoparticles has also been documented elsewhere [56]. The same explanation could similarly justify the positive cellular uptake of negatively charged rod-like FITC-CNC at pH 5 [15]. 

## 4. Conclusions

In conclusion, the present study has successfully synthesized stable spherical fluorescently-labelled CNC from oil palm empty fruit bunch CNF with a negative surface charge. For its low degree of cytotoxicity and poor non-specific internalization into normal and cancerous cell lines, FITC-CNC could potentially be developed as targeted nanocarrier for the delivery of anti-cancer drugs, DNA, or other macromolecules. 

## Figures and Tables

**Table 1:** Table summarising the zeta potential (ξ) and dispersity (Ð) of CNC and FITC-CNC.

Sample	Zeta Potential (ξ)	Dispersity (Ð)
CNC	−38.6 mV	0.703
FITC-CNC	−17.7 mV	0.371

## Supplementary Materials

The following are available online at https://www.mdpi.com/1996-1944/12/19/3251/s1. Figure S1: (a) AFM micrograph showing the co-existence of ribbon-shaped and spherical CNC. Histograms of (b) ribbon-shaped CNC length and (c) ribbon-shaped CNC diameter and their respective averages.
