# Supplementary material for: Characterization and Cellular Internalization of Spherical Cellulose Nanocrystals (CNC) into Normal and Cancerous Fibroblasts
Source: Materials (Basel). 2019 Oct 4;12(19):3251. doi: 10.3390/ma12193251 (PMC6803863; doi:10.3390/ma12193251)
Supplement: Supplementary file 1 [file materials-12-03251-s001.pdf]

# Supplementary materials:

# Characterization and Cellular Internalization of Spherical Cellulose Nanocrystals (CNC) into Normal and Cancerous Fibroblasts

**Nur Aima Hafiza Shazali [1], Noorzaileen Eileena Zaidi [2], Hidayah Ariffin [1,3], Luqman Chuah Abdullah [4], Ferial Ghaemi [1], Jafri Malin Abdullah [5], Ichiro Takashima [6] and Nik Mohd Afizan Nik Abd. Rahman [1,2,*]**

[1] Institute of Tropical Forestry and Forest Products (INTROP), Universiti Putra Malaysia, 43400 Serdang, Selangor, Malaysia
[2] Department of Cell and Molecular Biology, Faculty of Biotechnology and Biomolecular Sciences, Universiti Putra Malaysia, 43400 Serdang, Selangor, Malaysia
[3] Department of Bioprocess, Faculty of Biotechnology and Biomolecular Sciences, Universiti Putra Malaysia, 43400 Serdang, Selangor, Malaysia
[4] Department of Chemical and Environmental Engineering, Faculty of Engineering, Universiti Putra Malaysia, 43400 Serdang, Selangor, Malaysia
[5] Brain Mapping and Neuroinformatics Unit, Centre for Neuroscience Services and Research (P3Neuro), Health Campus, Universiti Sains Malaysia, 16150 Kubang Kerian, Kelantan, Malaysia
[6] Human Informatics Research Institute, National Institute of Advanced Industrial Science and Technology (AIST), Tsukuba, Ibaraki 305-8568, Japan
* Correspondence: m.afizan@upm.edu.my; +603-9769 1944

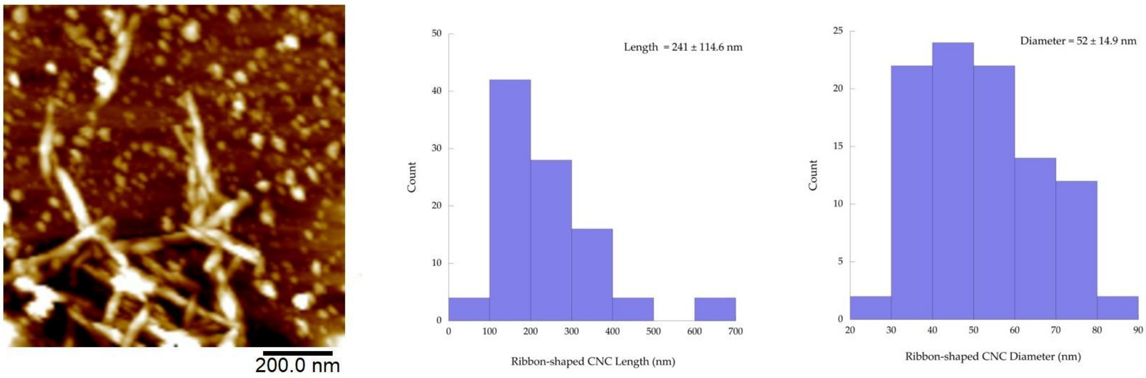


(a) (b) (c)

**Figure S1.** (**a**) AFM micrograph showing the co-existence of ribbon-shaped and spherical CNC. Histograms of (**b**) ribbon-shaped CNC length and (**c**) ribbon-shaped CNC diameter and their respective averages.
